# A mixed methods study of individual and organizational factors that affect implementation of interventions for children with autism in public schools

**DOI:** 10.1186/s13012-016-0501-8

**Published:** 2016-10-10

**Authors:** Jill Locke, Rinad S. Beidas, Steven Marcus, Aubyn Stahmer, Gregory A. Aarons, Aaron R. Lyon, Carolyn Cannuscio, Frances Barg, Shannon Dorsey, David S. Mandell

**Affiliations:** 1 Department of Speech and Hearing Sciences, University of Washington, 1417 NE 42nd St, Seattle, WA 98105 USA; 2 Department of Psychiatry, University of Pennsylvania Perelman School of Medicine, 3535 Market Street, 3rd floor, Philadelphia, PA 19104 USA; 3 Department of Social Policy and Practice, University of Pennsylvania Perelman School of Medicine, 3535 Market Street, 3rd floor, Philadelphia, PA 19104 USA; 4 Department of Psychiatry and Behavioral Sciences, University of California, Davis, 2825 50th Street, Sacramento, CA 95817 USA; 5 Department of Psychiatry, University of California San Diego, 9500 Gilman Drive, La Jolla, CA 92093 USA; 6 Department of Psychiatry and Behavioral Sciences, University of Washington, 6200 NE 74th St, Bldg. 29, St. 100, Seattle, WA 98115 USA; 7 Department of Family Medicine and Community Health, University of Pennsylvania Perelman School of Medicine, Philadelphia, PA USA; 8 Department of Psychology, University of Washington, Guthrie Hall, Seattle, WA 98195 USA

**Keywords:** Implementation, Attitudes, Organizational factors, Fidelity, Autism, Schools

## Abstract

**Background:**

The significant lifelong impairments associated with autism spectrum disorder (ASD), combined with the growing number of children diagnosed with ASD, have created urgency in improving school-based quality of care. Although many interventions have shown efficacy in university-based research, few have been effectively implemented and sustained in schools, the primary setting in which children with ASD receive services. Individual- and organizational-level factors have been shown to predict the implementation of evidence-based interventions (EBIs) for the prevention and treatment of other mental disorders in schools, and may be potential targets for implementation strategies in the successful use of autism EBIs in schools. The purpose of this study is to examine the individual- and organizational-level factors associated with the implementation of EBIs for children with ASD in public schools.

**Methods:**

We will apply the Domitrovich and colleagues (2008) framework that examines the influence of contextual factors (i.e., individual- and organizational-level factors) on intervention implementation in schools. We utilize mixed methods to quantitatively test whether the factors identified in the Domitrovich and colleagues (2008) framework are associated with the implementation of autism EBIs, and use qualitative methods to provide a more comprehensive understanding of the factors associated with successful implementation and sustainment of these interventions with the goal of tailoring implementation strategies.

**Discussion:**

The results of this study will provide an in-depth understanding of individual- and organizational-level factors that influence the successful implementation of EBIs for children with ASD in public schools. These data will inform potential implementation targets and tailoring of strategies that will help schools overcome barriers to implementation and ultimately improve the services and outcomes for children with ASD.

## Background

Autism spectrum disorder (ASD) is a complex developmental disorder characterized by difficulties with social communication and the presence of restricted and/or repetitive behaviors [1]. The Centers for Disease Control and Prevention (2014) estimate that 1 in 68 children in the USA have ASD [2]. Schools are the primary setting in which children with ASD receive intervention services, and the public education system has been greatly affected by the rising prevalence of ASD [3]. Schools are under increasing pressure to incorporate evidence-based interventions (EBIs) to meet the diverse needs of children with ASD [4]; however, to date, these interventions have not been effectively implemented and sustained in public schools [5, 6].

Implementing autism EBIs in schools is challenging, in part because of poor intervention-setting fit, defined as the interactions among: (1) the school context, (2) characteristics of the intervention, and (3) its intended users [7]. The autism spectrum includes children with a wide range of needs [8]. Given this diversity, autism EBIs often utilize multiple concurrent strategies that address academic, behavioral, and social outcomes. To a large extent, the degree to which teachers adopt, implement, and sustain EBIs depends on the fit of these strategies within the classroom structure [9]. The complexity and resource-intensive nature of autism EBIs make them difficult to implement in public schools, which often lack resources and trained staff [10–12]. Compounding this problem are two issues. First, due to the heterogeneity of students with ASD, teachers are expected to learn and integrate EBIs that entail multiple instructional strategies in their classrooms, often with little to no training on how to do so. It also may be unrealistic to use all of the components of autism EBIs at once. Implementing multiple instructional strategies may be more manageable when roll out is staggered or with appropriate support [13]. Second, school personnel generally receive little ongoing support in implementing EBIs [11, 14], and when they implement EBIs, generally, they do not achieve the same results as observed in controlled trials [15, 16]. Evidence from other disciplines suggests that EBIs often are not implemented and sustained in the way they were designed [17, 18]. Intervention-setting fit may play a role in sub-optimal implementation of autism EBIs in schools. Successful implementation may require tailoring implementation strategies to address the intervention setting, adapting aspects of the intervention or a combination of both [19–24]. Many of these issues are relevant to other mental health populations and service settings, but to date, they have not been examined within the context of autism EBIs in public schools.

A growing body of research in implementation of other evidence-based mental health interventions in school settings has identified a number of factors at the individual (e.g., attitudes toward EBIs) and organizational levels (e.g., organizational culture and climate, implementation climate, and leadership) that may affect implementation and sustainment of EBIs [7, 19–28]. At the individual level, implementers’ attitudes toward adoption of EBIs may hinder or facilitate implementation and these attitudes may vary by organizational context [29]. Organizational factors such as culture (behavioral expectations and norms that characterize the way work is done in a work environment) and implementation climate (staff beliefs on whether use of an innovation is expected, rewarded, and supported by their organizations) may be important determinants of a school’s capacity to provide appropriate support and resources necessary to implement EBIs and sustain change [30–35]. Leadership also may play a critical role in an organization’s readiness for change [35]. Based on our team’s prior research in other organizational settings and evidence from other disciplines, two types of leadership may be operative in schools, influencing the effectiveness of autism-related EBIs: (1) transformational leadership, the degree to which a leader (in our case, the principal) can inspire and motivate staff; and (2) transactional leadership, perceived support from a leader in the provision of incentives and rewards (e.g., praise, recognition, a title) [36–38]. Further, identifying specific leader behaviors may facilitate implementation of EBIs [39]. To date, these constructs have not been systematically studied with relation to the use of EBIs in public schools.

The participating school districts and the research team have long-standing relationships, which provide an ideal natural laboratory in which to systematically examine, on a large scale, implementation challenges of autism EBIs in a diverse set of schools. Specifically, we will examine predictors of implementation and sustainment of autism EBIs for children with ASD in public schools, with the ultimate goal of tailoring implementation strategies to increase uptake of autism EBIs. Specifically, the overall objectives of the proposed research are to: (1) quantitatively examine individual- and organizational-level factors as predictors of successful autism EBI implementation; (2) qualitatively examine the individual- and organizational-level factors associated with successful or unsuccessful implementation of autism EBIs; and (3) collaboratively tailor implementation strategies with our school partners to target individual- and organizational-level factors most amenable to change, and most important for successful implementation.

### Conceptual framework and approach

The conceptual framework for the proposed research is drawn from Domitrovich and colleagues (2008), who posit that implementation of EBIs in schools is influenced by a broad array of factors at the macro, school, and individual levels [40]. The macro level includes community factors such as policies and practices at the federal, state, or district level that may influence the quality of implementation within schools. School factors that influence intervention implementation include schools’ organizational functioning, policies within the buildings, resources available to support implementation, and organizational climate. The individual level includes factors associated with the implementer (i.e., teachers and classroom staff) that affect the quality of intervention implementation and includes professional and psychological characteristics and attitudes to the intervention [40]. This study will measure factors from the individual- (e.g., attitudes of EBIs) and organizational levels (e.g., organizational culture, implementation climate, leadership) to examine their association with the implementation and sustainment of autism EBIs in public schools. Although the Domitrovich and colleagues (2008) framework most closely relates to our work in schools, the constructs identified in this framework are broadly defined and the direction with which these factors are associated are unspecified [40]. Implementation research of autism EBIs in schools is in an early stage, and the relative contribution of individual- and organizational-level factors, particularly malleable factors of importance, is unknown. Given our experiences in schools and implementation research of other evidence-based mental health interventions, we will focus on the attitudes about EBIs at the individual level and organizational culture and climate, implementation climate, and leadership at the organizational level as potential levers of change. We hypothesize that (1) both individual- and organizational-level variables are directly associated; (2) both individual- and organizational-level variables will separately predict the implementation of autism EBIs; and (3) organizational characteristics will moderate the relationship between the individual-level variables and EBI implementation.

## Methods/design

Aim 1: Examine the effects of individual- and organizational-level factors on implementation and sustainment of EBIs for children with ASD.

This aim will quantitatively measure individual (e.g., attitudes of EBIs) and organizational (e.g., organizational culture and climate, implementation climate, leadership) characteristics that are drawn from the Domitrovich et al. (2008) framework as predictors of implementation and sustainment of autism EBIs [40].

### Participants

Participants will include 40 principals, 70 kindergarten-through-third-grade autism support teachers, and 70 classroom staff from an estimated 70 classrooms across 40 schools that use four EBIs (discrete trial training, pivotal response training, functional routines, and positive reinforcement) based on the principles of applied behavior analysis to address academic, behavioral, and social outcomes for children with ASD [41, 42].

### Procedure

The research team will first meet with the school district officials to obtain a list of kindergarten-through-third-grade autism support classrooms and schools. Subsequently, the research team will arrange meetings with the principal at each prospective school to discuss the research activities and obtain a letter of agreement to conduct research on their campus. All recruitment materials (e.g., informational handouts, flyers) will be distributed to the school, and the research team will meet with interested participants to inform them about the study and their role as a study participant, so they are able to make an informed decision regarding their participation. Once informed consent is obtained, the research team will ask principals, teachers, and classroom staff to complete all study measures (see below). Participants will be compensated with US$50 for their time.

### Measures

#### Dependent variable: fidelity

Program fidelity (i.e., adherence, dose, and competence) will be measured using an observer-rated fidelity checklist that examines four behavioral intervention strategies: discrete trial training, pivotal response training, functional routines, and positive reinforcement [43]. Adherence will be measured via direct observation using an implementation checklist and coded on a Likert scale from “0” (does not implement) to “4” (highly accurate). Dose will be monitored by direct observation and teacher report for each component and coded using a Likert scale ranging from “0” to “4” with the following criteria for each score: “0” (less than one time per week), “1” (one time per week), “2” (two to four times per week), “3” (one time per day), and “4” (two times per day) [44]. Competence will be measured via direct observation and coded for classroom preparedness and use of each evidence-based intervention strategy (discrete trial training, pivotal response training, functional routines, and positive reinforcement).

#### Independent variables

All measures except the Multifactor Leadership Questionnaire will be adapted in collaboration with the developers for use in the school context.

#### Attitudes toward EBIs

Attitudes about the use of EBIs will be measured using the Evidence-Based Practice Attitude Scale (EBPAS) [29, 45]. The EBPAS is a 15-item measure that assesses four general attitudes toward adoption of EBIs: appeal, requirements, openness, and divergence. Studies suggest moderate to good internal consistency for the EBPAS total score (Cronbach’s *α* = 0.76, 77, 0.79) and subscale reliabilities ranged from 0.67 to 0.91 [45–47].

#### Organizational culture and climate

Organizational culture and climate will be measured using the Organizational Social Context (OSC) for schools, a 105-item gold-standard measure that assesses organizational culture, climate, and work attitudes in public service settings [48]. The OSC is a reliable and valid measure that can be used to create profiles of organizational functioning. In partnership with the University of Tennessee, Knoxville, the research team will adapt the OSC to ensure its use is appropriate among special education teachers and staff in the school context.

#### Implementation climate

To measure implementation climate, we will use the Implementation Climate Scale (ICS), an 18-item rating scale that measures employees’ shared perceptions of the policies, practices, procedures, and behaviors that are expected, rewarded, and supported in order to facilitate effective EBI implementation [49]. The ICS has six subscales including (1) focus on EBIs, (2) educational support for EBIs, (3) recognition for EBIs, (4) rewards for EBIs, (5) selection for EBIs, and (6) selection for openness. The ICS is a psychometrically validated and reliable instrument (*α* = 0.81–0.91).

#### Leadership

To measure organizational leadership, we will use the Multifactor Leadership Questionnaire (MLQ) [36], a psychometrically validated measure that assesses transformational (i.e., intellectual stimulation, inspirational motivation, individual consideration, and idealized influence) and transactional (i.e., contingent reward) leadership. To identify specific leader behaviors, we will use the Implementation Leadership Scale (ILS), a 12-item rating scale with four subscales that assess the degree to which a leader is knowledgeable (deep understanding of EBI and implementation issues), supportive (support for EBI adoption/use), proactive (anticipating and addressing implementation challenges), and perseverant (consistent and responsive to challenges) in implementing EBIs [39]. The ILS also is a psychometrically validated and reliable instrument (*α* = 0.95–0.98) [39].

### Power analysis

We will have 80 % power with *α* = 0.05 to detect relatively small associations between components of the organizational model (Cohen’s *d* = 0.35). However, to account for nesting of staff within classrooms and schools, the sample size calculated under the assumption of simple random sampling must be deflated to account for the Intracluster Correlation Coefficient (ICC), a measure of the magnitude of relatedness of observations within versus between clusters. Assuming a conservative ICC of 0.2, we will ultimately be able to detect a moderate effect (Cohen’s *d* = 0.48).

### Analysis

The OSC, ICS, MLQ, and ILS will be aggregated across teachers and classrooms staff from each school to create organizational-level constructs if exploration of the data supports this (i.e., concordance between reporters). We will first examine the distribution of and correlations among variables. We will explore relevant subscales of all measure to understand the nuances of each construct. We will use linear regression with random effects for classroom and school to account for classrooms nested within schools to examine individual associations between each organizational-level factor (i.e., organizational culture, implementation climate, and leadership) and fidelity (i.e., adherence, dose, and quality of program delivery) as well as the relationships of those factors on individual-level factors (i.e., attitudes toward EBIs), and individual-level factors on each dimension of fidelity. We will use the bivariate associations to determine the most parsimonious adjusted model and observe the degree to which the magnitude of these associations changes.

Aim 2: Examine the implementation process for a subset of high performing and low-performing classrooms implementing an EBI for children with ASD.

We will (1) identify classrooms that are high- and low-performing in their implementation of autism EBIs and (2) study the characteristics and implementation processes of high- and low-performing classrooms. The ways in which implementation of the autism EBIs is staggered, delivered, and supported may be important for successful implementation. We will use qualitative methods (semi-structured interviews with 24 principals and 24 teachers) to understand the appropriateness and fit of the four autism EBIs from Aim 1 within the school context, and principals’ and teachers’ experiences and perspectives regarding the implementation process in a subset of classrooms that are either high (*n* = 12) or low performing (*n* = 12) based on their fidelity data from Aim 1. Qualitative analyses will allow us to conduct a detailed exploration of the intervention-setting fit, and observe similarities and differences in individual- and organizational-level factors among high- and low-performing classrooms.

### Participants

We will combine the multiple dimensions of fidelity data from Aim 1 into one total fidelity score. We will use the highest and lowest implementation fidelity scores from Aim 1 to identify 12 high-performing and 12 low-performing autism support classrooms. We anticipate that we will reach thematic saturation after completing approximately 12 interviews in each cell [50]. We will conduct iterative data reviews during the data collection process in order to assess when saturation has been achieved. We will recruit an anticipated total of 48 key stakeholders (24 principals and 24 teachers) from an estimated 24 classrooms in 24 schools. If newly recruited participants continue to contribute novel information, we will recruit additional participants until saturation has been reached.

### Procedures

Recruitment and informed consent procedures will follow those proposed in Aim 1. We will conduct semi-structured interviews individually with principals and teachers. The interviews will be audiotaped and conducted at schools at a convenient time for the participant, lasting approximately 45–60 min. As an incentive for participation, participants will be offered US$50 for their time.

#### Qualitative interviews

Based on the results from Aim 1, we will develop a semi-structured interview guide. We will examine the experiences and perspectives of principals and teachers in both high- and low-performing classrooms to understand the fit of the intervention components within the school setting as well as what may need to change with the implementation process. Using the organizational factors that broadly affect implementation of the autism EBIs examined in Aim 1, we will generate questions that explore the nuanced differences between high- and low-performing classrooms. We will draw from participants’ responses on the quantitative measures used in Aim 1 as a starting point and will gather more-detailed information on these constructs to determine which individual- and organizational-level factors are the most important for successful implementation and which are amenable to change. For example, we may ask participants about the implementation climate at their school, such as the ways in which principals facilitate or support teachers’ and classroom staff’s use of EBIs. Questions and interview guides will be carefully constructed to elicit clear information without assigning valence to the classroom performance. We also will ask questions about the roll out process of the autism EBIs and what scaffolds may be needed to support staff and schools in their use of autism EBIs that is feasible within the school context. The synthesis of this information will help us make an informed decision regarding what needs to be targeted in the intervention setting and implementation process to ensure successful implementation and sustainment occurs.

### Qualitative analysis

A transcription service will transcribe all interviews. The transcription service will comply with all HIPAA regulations and will be prohibited from duplicating or sharing any information from the audio recordings. Transcripts will be imported into NVivo QSR 11, a software package used to manage qualitative data. The development of the coding scheme will use a rigorous, systematic, transparent, iterative and integrated approach [51]. Coders will independently engage in line-by-line coding of an initial set of transcripts to identify recurring codes. They will meet as a group weekly to discuss recurring concepts and develop a codebook using an integrated approach to coding as certain a priori codes will be conceptualized during the interview guide development (i.e., deductive approach) and other codes will be developed through a close reading of the initial set of transcripts (i.e., inductive approach) [51]. These codes will provide a way of understanding the full range of individual- and organizational-level variables that affect the use of autism EBIs and also provide more in-depth data on the implementation process. The development of the codebook will include operational definitions of each code, examples of the code from the data, and when to use and not use the code. The coding scheme will be refined and then applied to the data to produce a descriptive analysis of each code, which will continue to be refined throughout the data analytic process [51]. After finalization of the codebook, coders will overlap on 20 % of randomly selected transcripts to determine inter-rater reliability. Final codes will be determined through a consensus process, where all reviewers will independently code all of the transcripts and meet to compare their coding to arrive at consensus judgments through open dialog [52–54]. Consensus coding is designed to capture data complexity, avoid errors, reduce groupthink, and circumvent some researcher biases. Inter-rater reliability between coders will be computed during the coding process and efforts will be made to achieve and maintain at least 80 % reliability using the inter-rater reliability function in NVivo [55]. A single team member will code the remainder of transcripts.

To take advantage of multiple perspectives (i.e., principals and teachers) and develop a complete understanding of the process underlying successful or unsuccessful implementation of autism EBIs, we will use the following sequential mixed methods design. We will collect quantitative data (Aim 1) prior to qualitative data (Aim 2); the function is of complementarity (we will use qualitative data to elaborate upon the quantitative findings to understand the implementation process as experienced by principals, teachers, and classroom staff QUAN→QUAL); and the process is connecting (we will have the qualitative data set build upon the quantitative data set) [56–59].

Aim 3: Tailor implementation strategies for the successful use of autism EBIs in schools.

Using data from Aims 1 and 2 and in collaboration with our school partners, we will tailor implementation strategies to address key factors at the individual- and organizational levels to aid in implementing and sustaining autism EBIs in public schools.

### Participants

We will recruit a subsample (*n* = 12) of participants from Aim 2 from three high- and three low-performing classrooms from six schools to serve on an advisory board to ensure the implementation strategies are tailored to support the use and sustainment of autism EBIs in school settings as well as feasible to use.

### Procedure

Although the exact targets of the implementation strategies will depend on the results of Aims 1 and 2, we anticipate that the proximal targets will be implementation climate and leadership. For example, we may actively attend to the school’s implementation climate by ensuring the use of autism EBIs is expected, supported, and rewarded through engaging dialogs that clarify and rally teachers and classroom staff, recognizing key staff through faculty meetings, praise, or rewards that are referred to as “meaningful but valueless”, in that they are important to the recipient but have no monetary value (e.g., certificates, titles that acknowledge expertise, etc.) [9, 60]; or we may improve implementation leadership by ensuring principals are knowledgeable (well versed in autism EBIs), supportive (recognize and support classroom staff’s use of autism EBIs), proactive (have a plan to implement autism EBIs), and perseverant in implementing autism EBIs (sustain use despite challenges) [39].

We will meet with principals and teachers three times during the school year. During the first meeting, we will present the conceptual model, discuss the results from Aims 1 and 2, outline areas of need as well as devise a plan for collaborative decision-making. Results will be presented in a number of ways including written description, graphs, and illustrations in order to facilitate communication with non-research stakeholders. During the next meeting, we will review the topic areas and all training materials (e.g., educational materials for staff, certificates of recognition, evaluation forms, schedule templates, etc.) as well as devise or adapt potential implementation strategies identified in Powell and colleagues (2015) with our school collaborators [61]. We will engage participants in frank discussion about potential implementation strategies to address the factors explored in Aims 1 and 2 and ask for their feedback to assess acceptability and feasibility. We will develop a measure to assess school personnel’s overall impressions, readability, usability, and strengths and weaknesses of the proposed implementation strategies and training materials. We will ask participants about the appropriateness and clarity of the descriptions of the strategies. Each item will be rated on a 1–5 Likert scale with response anchors tailored to the type of question. Space will be provided to make any additional comments. We will use these data to inform and guide subsequent revisions to the manual and meetings with school personnel. We will use the remaining meetings to review and adapt the implementation strategies and materials to maximize the potential fit of the strategy within the school context [62]. The meetings will last approximately 60–90 min, will be audiotaped, and conducted at schools at a convenient time for the participants. As an incentive for participation, each participant will be offered US$100 for each meeting.

### Trial status

The University of Washington and University of Pennsylvania as well as each participating school district’s Institutional Review Boards have approved the study procedures. At the time of submission of this manuscript (September, 2016), we have already enrolled principals, teachers, and classroom staff for data collection for Aims 1 and 2. We are continuing recruitment and data collection for Aims 1 and 2, which will begin in October, 2016.

## Discussion

### Innovation

This study contains three important innovations. First, this study will advance the field of implementation science by examining predictors of implementation and sustainment as a function of intervention-setting fit [7, 27, 28]. With complex autism EBIs, successful implementation may be related to the implementation process as tailored for the organizational context rather than as a function of the core components of the intervention. Second, this study will adapt several existing gold-standard measures of individual- and organizational-level factors for use in school settings that may broaden the field. Lastly, this will be one of the first studies to prospectively examine predictors of implementation and sustainment of EBIs for children with ASD in public schools. The increased prevalence of ASD has made improving the implementation of EBIs for children with ASD in public schools a priority among funders, advocates, educators, policy makers, and researchers [9]. If successful, this study has the potential to improve the quality of care for many children with ASD in the schools that serve a disproportionately large number of them [63].

### Limitations

The proposed study is one of the first to prospectively examine individual- and organizational-level predictors of implementation and sustainment of EBIs for children with ASD in public schools, though it is not without its limitations. The Domitrovich and colleagues (2008) framework accounts for macro-level factors, which encompasses district-level variables that may predict successful implementation, which we will not measure in this study [40]. Given schools’ autonomy in the districts with which this study will be conducted, district-level policies may be distally related to implementation. These districts have mandated and provided substantial training in several autism EBIs to their K-3 autism support classrooms, which creates a level policy environment in which to examine implementation issues, yet there is still significant variability in implementation and sustainment in these schools. Additionally, we understand that the proposed organizational measures are typically designed for aggregating a number of raters in one setting. However, this is difficult to achieve in classrooms in which there is only one teacher and schools with one leader (i.e., principal). To address this challenge, we will use multiple raters within the entire school (i.e., teachers and other classroom staff).

### Impact

The significant lifelong impairments associated with ASD, combined with the growing number of children diagnosed with this disorder, create a sense of urgency in improving school-based services. Our collaboration with our school partners provides a natural laboratory in which to examine critical issues related to implementation. The proposed research activities will result in an in-depth understanding of individual- and organizational-level variables influencing implementation of EBIs for children with ASD in public schools, and a set of tailored implementation strategies adapted in collaboration with schools that, if successful, will help schools implement EBIs that address academic, behavioral, and social outcomes for children with ASD. More broadly, the results of this study also may be generalized to other populations or settings.

## Abbreviations

ASD: Autism spectrum disorder
EBI: Evidence-based intervention
EBPAS: Evidence-based practice attitude scale
ICS: Implementation climate scale
ILS: Implementation leadership scale
OSC: Organizational Social Context
